# Effect of processing and soybean cultivar on *natto* quality using response surface methodology

**DOI:** 10.1002/fsn3.848

**Published:** 2018-11-25

**Authors:** Mahalaxmi Pradhananga

**Affiliations:** ^1^ Central Campus of Technology Tribhuvan University Dharan Nepal

**Keywords:** bacillus, fermentation, natto, soybean

## Abstract

In an attempt to commercialize the traditional technology of fermenting soybean into *natto* on laboratory scale using three locally available soybean varieties, that is, white, black, and brown, response surface methodology (RSM) was used to determine the optimum combination of two factors, that is, the effect of steaming time (20–50 min) and fermentation time (12–48 hr). Thirteen samples from each variety were formulated which were packed in low‐density polyethylene for LDPE using the isolated culture from the *natto* sample and incubated at 37°C, and the sensory data were analyzed by using Design Expert (RSM). All the responses (taste, hardness, thread/stickiness, and overall acceptance) were significantly (*p* < 0.05) affected by the two variables except appearance for all three varieties of *natto* prepared. The optimum combinations of steaming time (min) and fermentation time (hr) were found for white soybean *natto* (33.4 min and 34.5 hr), for black soybean *natto* (34.7 min and 30.9 hr), and for brown soybean *natto* (33.2 min and 34.9 hr), respectively. The proximate composition of soybean and best three formulated *natto* obtained after sensory evaluation from 13 samples of each variety were studied, that is, moisture, crude protein, pH, calcium, and iron on dry basis found to have increased, whereas carbohydrate and crude fiber found to have slightly decreased, but crude fat and ash found to be almost equal on three varieties than the raw soybean used. Microbiologically, the product was hygienic and safe as coliform and salmonella were not detected.

## 1 INTRODUCTION

*Natto* is one of the few products in which bacteria predominate during fermentation. The responsible bacterium has been identified as *Bacillus natto*, an aerobic Gram‐positive rod‐shaped aerobic spore former closely related to *Bacilllus subtilis*. The *natto* is a Japanese fermented soybean food that has a characteristic ammonia odor, contains fatty acids and musty flavor, and has a slimy appearance being covered with viscous and sticky polymers of glutamic acid (Hosoi & Kiuchi, 2003). *B. subtilis* fermentation of soaked and cooked soybeans results in proteolysis of soy polypeptides, altering its digestibility, taste, and flavor, as well as improving the protein quality. Besides, it inactivates antinutritional factors, removes indigestible oligosaccharides, and increases isoflavone, proteolytic enzymes, and phytosterols that can make difference in human health (Shrestha, Dahal, & Ndungutse, 2010).

*Natto* has nearly double the calcium and far more vitamin E. *Natto*, the “perfect food,” produces 18 valuable amino acids and an enzyme *natto*kinase that may challenge the pharmaceutical industry's best “blood‐clot busters” (Holsworth, 2002). Compared to boiled soybeans, *natto* is a powerhouse source of vitamin K, particularly K_2_ (rare in other foods). Vitamin K is known to be antagonistic to warfarin, while it plays an important role in blood coagulation and osteogenesis (Sumi, Hamada, Nakanishi, & Hiratani, 1990).

Soybean (*Glycine max* L.) is a leguminous crop called as king of legume, the “miracle crop” and “gold from the soil” (Smith & Circle, 1978). In Nepali, it is called Bhatmas (Katawal, 1984). The most dominant varieties of soybean in Nepal are of white, brown, gray, and black colors. It has different local name depending on the varieties, color of seeds, and locations such as Nepale, Hardi, Saathiya, Darmali, Maily, Kalo, and Seto (Shrestha, 1989).

Traditional fermented food has a tremendous potential for alleviating protein energy malnutrition particularly in third world, as they are nutritious, cheap, and easy to prepare*. Natto* has been consumed by the Japanese for more than hundreds of years, proving that *natto* is a very safe food (Hosoi & Kiuchi, 2003). It has been prepared easily due to its pure culture availability, and thus, the fermentation of soybean has been easily performed. As I have seen that soybean fermentation sometimes gets wasted due to growth of undesirable microorganisms, thus to prevent and to make the product safe and hygienic to be consumed with easy preparation method, this study will help the people for making the *natto* from varieties of soybean as per the availability with pure culture. The production of *natto* with pure culture can increase market and consumption rate. Shrestha (1997) has concluded that *natto* was good in quality than kinema as a food.

The quality of traditionally fermented foods, however, is low that warrants the use of pure culture to improve the quality. The nutritive qualities of *B. subtilis*‐fermented soybeans can best be utilized by developing countries. The use of pure culture helps in continuous and consistent quality of *natto* production. This study used pure *B. subtilis* (*Natto*) (isolated or from stock culture) as a primary inoculum to produce hygienic, safe, and better quality fermented soy products. In order to increase usefulness and acceptability, diversification of the products has long been realized. However, fermented products are still largely produced by traditional method; therefore, it was suggested to establish control measures and follow good manufacturing practice for people involved in its production and commercialization to ensure its safety.

## 2 MATERIALS AND METHODS

Soybean was cleaned, then soaked in water for about 12 hr and was drained completely. Procurement of *natto*: Takano Foods Co., Ltd (Okame *natto* Takanofuzu Ltd.) were used for isolation of *Bacillus natto (Bacillus Subtilis)*. Optimization of the parameters, (a) steaming time (20–50 min) and (b) fermentation time (12–48 hr), was performed on the three varieties using response surface methodology (RSM). Thirteen samples were prepared on each variety and were subjected for sensory evaluation to optimize the product. Chemical analysis of three best obtained products was carried out from 13 products each after sensory evaluation.

### 2.1 Preservation of *Natto* (low‐temperature storage)

#### 2.1.1 Methodology: isolation of pure culture of *Bacillus subtilis*

*Bacillus subtilis* was isolated from the sample of *natto* using two methods: suspension of aseptically weighed 10 g of *natto* sample in two test tubes in method I and method II with 9 ml sterile distilled water. In the method II, the test tube was placed in water bath set at 80°C for 10 min (Harrigan, 1998). A loopful of sample was taken, and streaking was done on nutrient agar plate. The plates were incubated at 37–40°C for 24–48 hr in incubator in inverted position. Streaking and incubation process were repeated for obtaining characteristic white, circular, and spreading nature colony.

#### 2.1.2 Colonial test of *Bacillus subtilis*

It was performed as described by Pelczar, Chan, and Krieg (1993). Biochemical test includes catalase test, Gram reaction, growth on 7% NaCl, mannitol test, and anaerobic growth by the method described in Bergey's Manual of Determinative Bacteriology (Cowan, Holt, & Liston, 1974). After the confirmation of *B. subtilis* colonies, the pure culture of *B. subtilis* was maintained by streaking and incubation at 37°C for 24 hr on nutrient agar slant, then *B. subtilis* from NA slant were transferred aseptically to nutrient broth, and incubation was done at 37°C for 24 hr. Then, it was preserved under refrigerated condition. Successive subculturing was carried out in every 15 days (Harrigan, 1998).

### 2.2 Preparation of starter culture or seed culture

**Figure 1:**
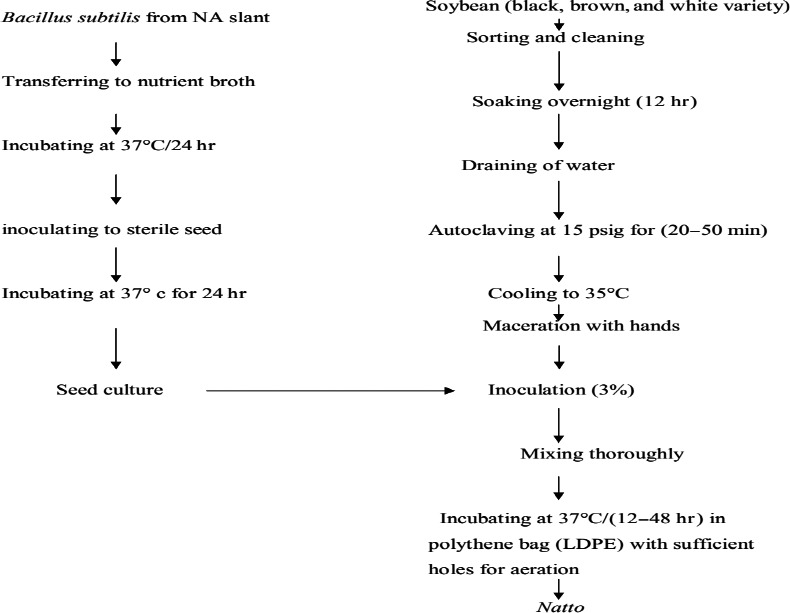
Preparation of Itohiki natto

One kilogram of each variety of soybean was cooked in autoclave. Soybean seeds were macerated and inoculated with culture broth, and incubation was done at 37°C for 24 hr and was used as culture. *Natto* sample was prepared from three varieties of soybean by the use of pure culture isolation. The flowchart for the preparation of Itohiki natto is shown in Figure 1.

#### 2.2.1 Optimization of process

**Figure 2:**
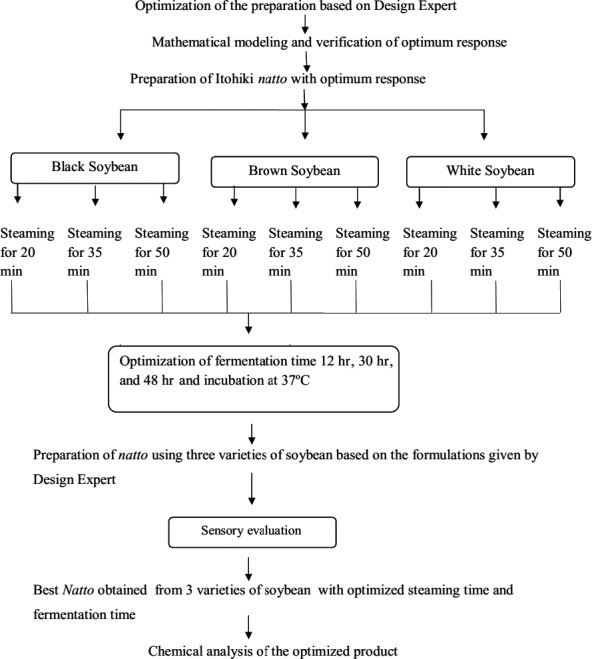
Recapitulative diagram of the experimental procedure

The recapitulative diagram of the experimental procedure is given in Figure 2.

Response surface methodology was used to determine the optimum conditions of two factors: steaming time (X1) and fermentation time (X2) (Montgomery, 2001). Each variable to be optimized was coded at three levels: −1, 0, and 1 (Table 1). A total of 13 runs each, for three varieties of soybean (black, white, and brown), were based on a central composite design of the 2nd order (Table 2). Experimental design in coded form is shown in Table 3.

**Table 1:** Independent variables and their coded levels and actual values used for optimization

Independent variables	Uncoded	Levels in coded form		
		−1	0	+1
Steaming time	X_1_	20	35	50
Fermentation time	X_2_	12	30	48

**Table 2:** Two‐factor, three‐level central composite design used for RSM

Standard order	Steaming time	Fermentation time	Steaming time	Fermentation time
	Uncoded		Coded	
	X_1_ Steaming time	X_2_ Fermentation time	X_1_	X_2_
1	20	12	−1	−1
2	50	12	1	−1
3	20	48	−1	1
4	50	48	1	1
5	20	30	−1	0
6	50	30	1	0
7	35	12	0	−1
8	35	30	0	1
9	35	30	0	0
10	35	30	0	0
11	35	30	0	0
12	35	30	0	0
13	35	30	0	0

**Table 3:** Experimental design in coded form for response surface analysis

Coded variables		Combinations	Replications	No. of. Expt
X_1_	X_2_			
±1	±1	4	1	4
±1	0	2	1	2
0	±1	2	1	2
0	0	1	5	5

Independent variable:

- Raw material: Soybean (black, white, and brown)
- Steaming time: (20–50 min) at 121°C
- Fermentation time (hour): (12–48 hr) at 37°C

Response variables: sensory evaluation.

The experiment was conducted with three replications. The experimental data were analyzed using ANOVA. The means were compared by L.S.D. method at 5% level of significance. Sensory data of the *natto* samples were evaluated using hedonic rating test as response variables and choose the best three *natto* from three varieties of soybean (Ranganna, 2000).

### 2.3 Analytical method

Physical properties such as color, shape, and surface were determined by visual inspection method. Length and breadth of soybean seed were measured by micrometer screw gauge. Bulk density was determined by calculating the weight of soybean seeds using a 100‐cc measuring cylinder.

### 2.4 Analysis of chemical component of soybean and *natto*

Moisture content, crude fat, crude protein, total ash content, and crude fiber content were determined (Ranganna, 2000). For *natto*, the pH was determined directly by inserting into fresh *natto* (method cited from Nepali, 2007). The carbohydrate content was determined by difference method by Shrestha (1997). Mineral (iron and calcium) content was determined by AOAC (2005).

### 2.5 Microbiological analysis

Total coliform and total plate count (TPC) were determined by Harrigan (1998). Yeast and mold count was determined by KC and Rai (2007). *Salmonella* spp. was determined by Varadaraj (1993).

## 3 RESULTS AND DISCUSSION

### 3.1 Physical properties of soybeans

The physical properties of soybean collected locally from Dharan were determined, and the results are shown in Table 4.

**Table 4:** Physicochemical properties of soybeans^a^

Properties	White soybean	Black soybean	Brown soybean
Color	Yellowish white	Black	Brown
Shape	Oblong and elliptical	Oblong	Oblong and spherical
Surface	Glossy	Smooth and glossy	Smooth
*L*/*b* ratio	0.83 (0.02)	1.12 (0.03)	1.04 (0.04)
Bulk density (kg/m^3^)	612.42 (3.58)	778.24 (4.34)	739.94 (2.74)
1,000 kernel	169.01 (3.15)	252.42 (5.25)	184.53 (6.67)

Values are the means of triplicates. Figures in the parentheses are the standard deviations.

The *L*/*b* ratio gives the idea about shape (sphericity) of the seed (Shrestha, 1997). The mean *L*/*b* ratio of white, brown, and black varieties of soybeans was found to be 0.83 ± 0.02, 1.04 ± 0.07, and 1.12 ± 0.03, respectively. The mean L/b ratio was reported to be 1.18 for black soybean variety (Shih, Yang, & Kuo, 2002). The *L*/*b* ratio for white and brown varieties was lesser than that obtained by Nepali (2007); however, for the black varieties, the ratio was in consonance. The bulk density observed for white soybean, brown soybean, and black soybean was 612.42, 739.94, and 778.24 kg/m^3^, respectively. The bulk density of white soybean was quite higher than that obtained by Shrestha (1989) and Dhungel (2000). The higher the 1,000 kernel weight is, the greater the size of the seed is 1,000 kernel weight for black soybean was 252.42 ± 5.25 g, for white soybean was 169.01 ± 3.15 g, and for brown soybean was 184.53 g. This indicates that black soybean is greater in its size than other two varieties. The value for black soybean was slightly higher (190 g), as determined by Dhungel (2000) and Nepali (2007), while regarding white and brown varieties, the values were far lesser.

### 3.2 Test of *Bacillus subtilis*

Bacillus *natto*, that is, *B. subtilis*, was isolated from the sample of *natto* brought from Japan. The test of identification was performed and is shown in Table 5.

**Table 5:** Test of identification of *Bacillus subtilis*

Parameter	Observation	Biochemical test	
Colonial test			
Color	White	Catalase test	Positive
Shape	Circular	Gram reaction	Positive
Length	2.0437 μm	Growth on 7% Nacl	Positive
Breadth	0.7846 μm	Mannitol test	Positive
		Anaerobic growth	Negative

White, circular, or round colonies of *B. subtilis* were observed on nutrient agar media. Length and breadth of the organism determined with the help of ocular micrometer were 2.0437 and 0.7846 μm, respectively. Gordon, Haynes, and Pang (1973) reported the length of *B. subtilis* to be in the range of 2–3 μm and that of breadth in the range of 0.7–0.8 μm. So the above dimension obtained for the organism was comparatively in the range given by Gordon et al. (1973).

The entire biochemical tests (Table 5) were done to check the confirmation of the organism as *B. subtilis* (*natto*). Anaerobic growth was not shown. The systematic characteristics of *Bacilli natto* correspond very well to those of *B. subtilis* as described in the seventh and later editions of Bergey's Manual of Determinative Bacteriology (8th edition). As described by Bergey's Manual of Determinative Bacteriology (8th edition), *B. subtilis* (*natto*) are Gram‐positive and catalase‐positive, show growth on 7% NaCl, give positive mannitol test, and show no growth in anaerobic agar. Both colonial test and biochemical tests confirmed the isolated organism as *B. subtilis* (*natto*).

### 3.3 Chemical analysis of soybean

*Natto* was prepared from three varieties of soybeans which were analyzed, and proximate compositions of soybeans were comparatively equal to that given in nutrient content of Nepalese food (1986). The proximate composition of soybeans was also determined, and the results are given in Table 6.

**Table 6:** Proximate constituents of different soybean varieties (values are in dry basis)^a^

Proximate constituents	White (g/100 g)	Brown (g/100 g)	Black (g/100 g)
Moisture	8.16 (0.86)	9.69 (0.04)	10.24 (0.86)
Crude protein	39.01 (2.4)	40.01 (3.1)	38.1 (1.1)
Crude fat	17.14 (0.68)	18.12 (0.9)	17.71 (0.6)
Crude fiber	4.56 (0.2)	3.86 (0.32)	4.38 (0.15)
Ash	5.12 (0.25)	5.42 (0.55)	5.66 (0.26)
Carbohydrates	34.17 (5.95)	32.59 (6.45)	34.15 (5.46)
pH	6.51 (0.005)	6.48 (0.01)	6.22 (0.001)
Calcium (mg)	220 (0.001)	235 (0.05)	330 (0.5)
Iron (mg)	8.8 (0.06)	10.6 (0.05)	9.6 (0.001)

Values are the means of triplicates. Figures in the parentheses are the standard deviations.

The analytical results showed that the values of moisture content obtained in this study were lesser than those obtained by Dawadi (1985), that is, 11.5%. The moisture content of white, brown, and black soybean varieties was reported to be 9.15, 9.89, and 10.14%, respectively (Nepali, 2007), which is similar to the results obtained in this experiment. The protein, fat, fiber, and ash contents were similar to the results obtained by Nepali (2007).

### 3.4 Optimization of formulation of *natto*

#### 3.4.1 Effect of process variables on overall acceptance (OA) of white soybean *natto*

**Figure 3:**
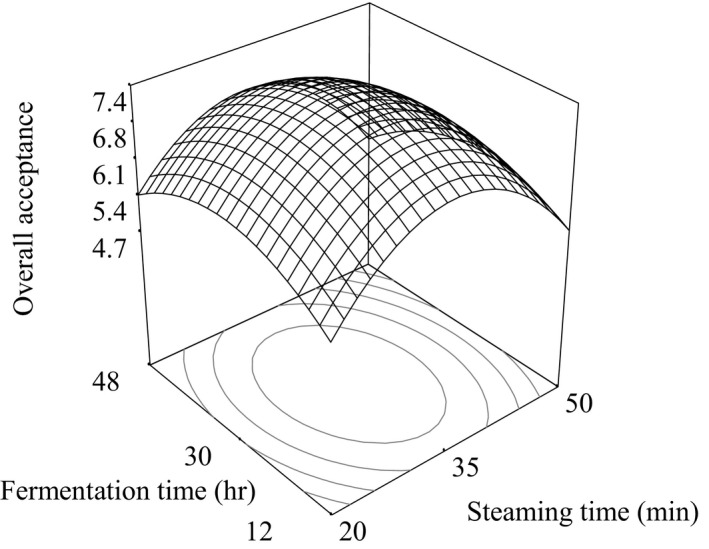
Response surface plots for the overall acceptance (OA) of white soybean *natto* as a function of steaming time and fermentation

Regression model fitted to experimental results of overall acceptability showed that model *F‐*value 9.69 was significant (*p* < 0.05). The chance of large model *F*‐value due to noise was only 0.48%. Lack‐of‐fit *F*‐value of 0.86 was not significant (*p* > 0.05). The chance of large lack‐of‐fit *F*‐value due to noise was 0.15%. The coefficient of determination *R* ^2^ was found to be 0.8738, indicating that 87.38% of the variability of the response could be explained by the model. The adjusted *R* ^2^ was found to be 0.7836. Adequate precision was 7.763, which is greater than 4. Considering all the above criteria, the model (Equation (1)) was selected from regression analysis in terms of coded values of the variables which is as follows:

(1) $$\text{OA}=7.43-0.2A-0.05B-0.15AB-1.52A^{2}-0.77B^{2}$$

where *A* and *B* are the coded values of steaming time (20–50 min) and fermentation time (12–48 hr), respectively. Figure 3 shows the response surface plot for the overall acceptance (OA) of *natto*.

The analysis of variance of Equation (1) showed the overall acceptability of the 13 samples of brown soybean *natto* did not had significant effect (*p* > 0.05) on steaming time (*A*), fermentation time (*B*), and interaction term (*AB*). But the quadratic terms of steaming time (*A* ^2^) and fermentation time (*B* ^2^) had significant (p < 0.05) effect.

#### 3.4.2 Effect of process variables on overall acceptance (OA) of black soybean

**Figure 4:**
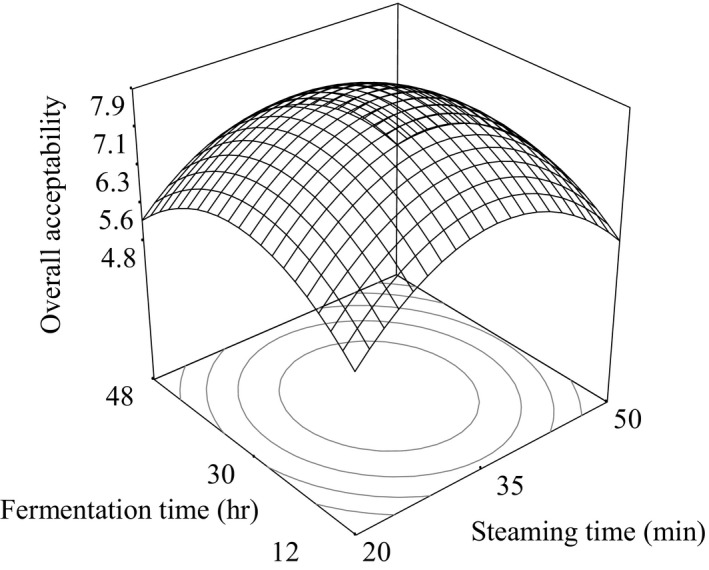
Response surface plot for the overall acceptance (OA) of black soybean *natto* as a function of steaming time and fermentation time

Regression model fitted to experimental results of overall acceptability showed that model *F*‐value 36.39 was significant (*p* < 0.05). The chance of large model *F*‐value due to noise was only 0.01%. Lack‐of‐fit *F*‐value of 31.22 was significant (*p* < 0.05). The chance of large lack‐of‐fit *F*‐value due to noise was 0.31%. The coefficient of determination *R* ^2^ was found to be 0.9629, indicating that 96.29% of the variability of the response could be explained by the model. The adjusted *R* ^2^ was found to be 0.9365. Adequate precision was 14.54 which is greater than 4, and hence, this model may be used to navigate the design space. Considering all the above criteria, the model (Equation (2)) was selected in terms of coded values of the variables which is as follows:

(2) $$\text{OA}=7.86+0.05A+0.1B+0.05AB-1.27A^{2}-0.82B^{2}$$

where *A* and *B* are the coded values of steaming time (20–50 min) and fermentation time (12–48 hr), respectively. Figure 4 shows the response surface plots for the OA of black soybean *natto*.

The analysis of variance of Equation (2) showed the overall acceptability of the 13 samples of black soybean *natto* did not had significant effect (*p* > 0.05) on steaming time (*A*), fermentation time (*B*), and interaction term (*AB*). The quadratic terms of steaming time (*A* ^2^) and fermentation time (*B* ^2^) had significant (*p* < 0.05) effect. The overall acceptance of the black soybean *natto* in 13 samples was increased as shown in Figure 4 to the formulations made in the range (34–35 min) steaming time and (30–31 hr) fermentation times, but it was then decreased with the increase of both parameters.

#### 3.4.3 Effect of process variables on overall acceptance (OA) of brown soybean

**Figure 5:**
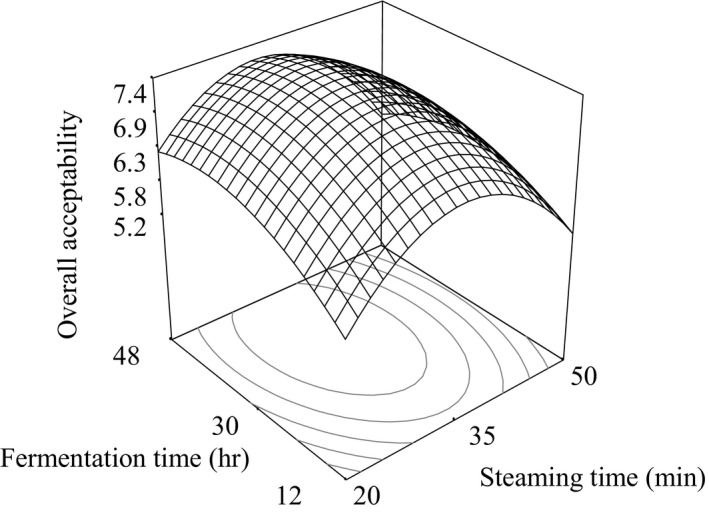
Response surface plots for the overall acceptance (OA) of brown soybean *natto* as a function of steaming time and fermentation time

Regression model fitted to experimental results of overall acceptability shows that model *F*‐value 3.99 was significant (*p* < 0.05). The chance of large model *F*‐value due to noise was only 4.94%. Lack‐of‐fit *F*‐value of 76.12 was significant (*p* < 0.05). The chance of large lack‐of‐fit *F*‐value due to noise was 0.06%. Significant lack of fit is bad. The coefficient of determination *R* ^2^ was found to be 0.7403, indicating that 74.03% of the variability of the response could be explained by the model. The adjusted *R* ^2^ was found to be 0.5548. Adequate precision was 4.764 which were greater than 4, and hence, this model may be used to navigate the design space. The analysis of variance of Equation (3) showed the overall acceptability of the 13 samples of brown soybean *natto* did not had significant effect (*p* > 0.05) on steaming time (*A*) and fermentation time (*B*). The quadratic terms of steaming time (*A* ^2^) had significant (*p* < 0.05) effect, but fermentation time (*B* ^2^) had no significant (*p* > 0.05) effect. The interaction term did not have significant (*p* > 0.05) effect. Considering all the above criteria, the model (Equation (3)) was selected from regression analysis in terms of coded values of the variables which is as follows:

(3) $$\text{OA}=7.36-0.22A+0.3B-0.17AB-1.27A^{2}-0.52B^{2}$$

where *A* and *B* are the coded values of steaming time (20–50 min) and fermentation time (12–48 hr), respectively. The overall acceptance of the brown soybean *natto* in 13 samples was increased as shown in Figure 5 to the formulations made upto in the range of (33–34 min) steaming time and (34–35 hr) fermentation times, but it was then decreased with the increase of both parameters. Figure 5 shows the response surface plots for the OA of brown soybean *natto*.

### 3.5 Solution of the experiment

RSM predicted the solution of the experiment for white soybean *natto*, black soybean *natto*, and brown soybean *natto* which included 33.4, 34.7, and 33.2 min steaming time, respectively, and 34.5 hr, 30.9 hr, and 34.9 fermentation time, respectively, and was prepared as recommended by design expert. Thus, the actual value of deviation was below 10% which is acceptable for all the sensory parameters.

Therefore, the optimum condition of three varieties of soybean *natto* for steaming time was found to be in the range (33–35 min) and for fermentation time showed optimum in the range (30–35 hr). Shrestha et al. (2010) used soaked beans, which were autoclaved at 121°C for 35 min, cooled to 30–35°C, inoculated with pure culture, and fermented at 37°C for 48 hr at 85% RH. Shih, Yang, and Kuo (2009) have reported that optimization of fermentation time in the range (12–36 hr) for producing black soybean *natto* and found the optimum result between (30–33 hr). For the obtained result is supported by Matsumoto, Akimoto, and Imai (1995) investigated that from sensory evaluation of the final *natto* products, they concluded that the optimum steaming time for soybeans was 30–40 min at 1.5 kg/cm^2^ pressure. Steaming for 30–40 min was found to be optimal for the production of appropriately aged *natto*; steaming for longer than 40 min yielded excessively aged *natto*.

**Figure 6:**
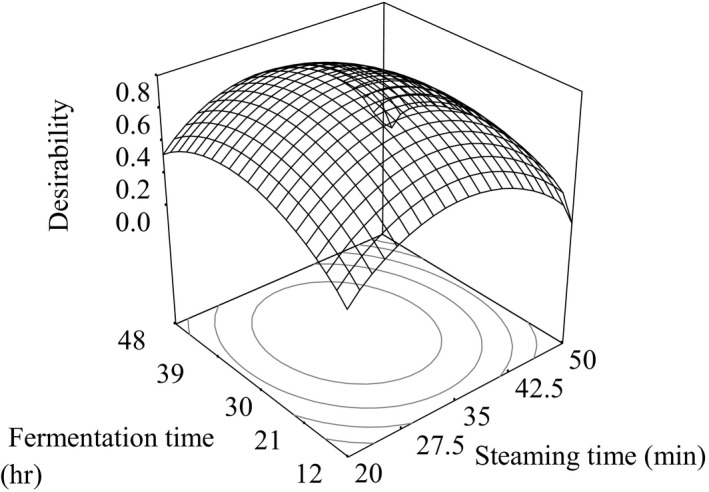
Response surface plot for desirability of white soybean *natto*

**Figure 7:**
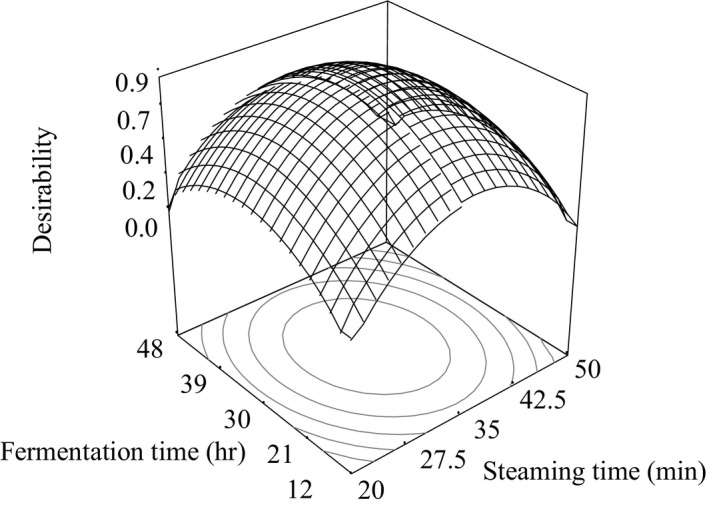
Response surface plot for desirability of black soybean *natto*

**Figure 8:**
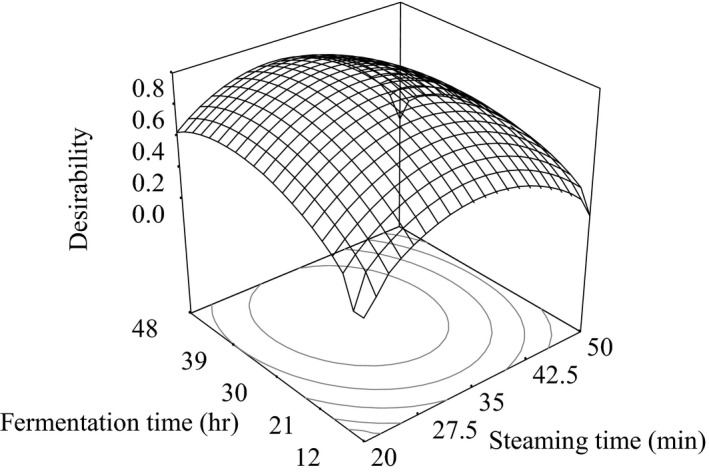
Response surface plot for desirability of brown soybean *natto* as a function of fermentation time and steaming time

Response surface plot for desirability of white, black, and brown soybean *natto*, respectively, as a function of fermentation time and steaming time is shown in Figures 6, 7, 8.

The responses predicted for the solution of the experiment are shown in Table 7.

**Table 7:** Solution of the experiment

Parameters	White	Black	Brown
Steaming time	33.4	34.7	33.2
Fermentation time	34.5	30.9	34.9
Desirability	0.852	0.868	0.819

### 3.6 Verification of result

The suitability of the model developed for predicting the optimum response values was tested using the second sensory evaluation and was also used to validate experimental and predicted values of the responses as shown in Table 8.

**Table 8:** Percentage deviation table of predicted and actual value

Variety	Responses	Appearance	Taste	Hardness	Thread/stickiness	Overall acceptance
White	Predicted	7	7.25	6.8	7.23	7.38
	Actual	7.2	7.9	7.2	7.5	7.5
	%Deviation	2.86	8.96	5.9	3.73	1.63
Black	Predicted	6.88	7.86	7.96	7.86	7.88
	Actual	7	8	8.2	8.2	8.3
	%Deviation	1.74	1.78	3.01	4.33	5.33
Brown	Predicted	6.8	7.29	6.8	7.27	7.42
	Actual	7.2	7.5	7.5	7.5	7.6
	%Deviation	5.9	2.9	10.29	3.16	2.43

The value of deviation about 10% is acceptable. The percentage deviation of the hardness is 10.29%, which is little greater than the acceptable value.

White soybean *natto*, black soybean *natto*, and brown soybean *natto* showed that the parameters steaming time and fermentation time had no significant difference (*p* > 0.05) in appearance, whereas they showed significant difference (*p* < 0.05) in taste, hardness, thread/stickiness, and overall acceptance through sensory evaluation. The optimum combinations of steaming time and fermentation time among the 13 samples each for three varieties were as follows: For white soybean natto as suggested by RSM, it was found as 33.4 min and 34.5 hr, respectively; for black variety, it was found as 34.7 min and 30.9 hr; and for brown soybean natto, it was found as 33.2 min and 34.9 hr and was prepared as suggested. Thus, the actual value of deviation was below 10% which is acceptable for all the sensory parameters for white and black soybean *natto*, but for brown soybean *natto*, other parameters were acceptable except for hardness which was 10.29%.

### 3.7 Analysis of the product

The chemical analysis of the optimized product of three soybean varieties *Natto* was done. The analytical result of the proximate composition of the *natto* is given in Table 9.

**Table 9:** Chemical analysis of *natto* samples

Parameter	White *natto*	Black *natto*	Brown *natto*
Moisture (%)	63.67 ± 0.47	60.5 ± 0.5	64.9 ± 0.173
Crude protein (% db)	48.22 ± 0.121	47.54 ± 0.451	49.6 ± 0.273
Crude fat (% db)	18.1 ± 0.431	18.01 ± 0.519	18.4 ± 0.415
Ash (% db)	5 ± 0.155	5.56 ± 0.123	5.37 ± 0.141
Crude fiber (% db)	3.49 ± 0.062	3.55 ± 0.135	3.35 ± 0.091
Carbohydrate (% db)	25.19 ± 0.192	25.34 ± 0.151	23.28 ± 0.119
pH (fresh)	7.6 ± 0.005	7.9 ± 0.01	7.4 ± 0.001
Calcium (mg/100 g)	322 ± 0.01	416 ± 0.01	340 ± 0.1
Iron (mg/100 g)	10.6 ± 0.005	11.6 ± 0.001	12.4 ± 0.01
Total plate count (cfu/g)	254	350	316
Yeast and mold count (cfu/g)	44	90	85
Coliform count	ND	ND	ND
Salmonella	ND	ND	ND

Values are the means of triplicates. Figures in the parentheses are the standard deviation.

ND: not detected.

The increase in moisture content with fermentation may be due to the high‐humidity (95% RH) environment to maintain its humidity, the hydrolytic decomposition of the fermenting substrate by *B*. *natto*, and the sticky quality of materials produced by fermentation covering the fermented black soybeans, which prevent evaporation of moisture (Hu et al., 2010). In a study of suitability of soybean varieties grown in Ibaraki prefecture (Japan) for *natto* production, Taira (1983) reported that the average of moisture content after steaming was 59.5% (58.1%–60.6%).

Protein content in all the varieties of *natto* was found to increase than that of raw material (Table 6). The increase in crude protein content might be due to the increased amount of nitrogen, as Hayashi (1974) has observed the nitrogen‐fixing capacity of *B. subtilis*. However, most of the literature have reported a general increase in protein content during *B. subtilis* fermentation, for example, 44.1%–46.2% (traditional) and 45.1% (pure culture) fermentation on dry basis (Shrestha & Noomhorm, 2001); 35.7%–40.3% (Hu et al., 2010); and overall 7% increase (Nikkuni et al., 1995) and 14% increase over raw soybeans (Hayashi, 1974). It is believed that increase in protein content is associated with the increase in microbial synthesis of protein or enzymes or rearrangements of compounds followed by degradation of other compounds (Hu et al., 2010). The crude fat content of all the varieties of *natto* was almost equal to their respective raw materials (Table 6). This indicates that *B. natto* does not have lipase‐producing ability or does not secrete it. Literature data showed *B. subtilis* fermentation causes very little change in lipid content of soybeans (Hu et al., 2010).

Not much change in ash content was observed in all the varieties of *natto*. It lied almost in between that of the raw materials (Table 6). The effect of fermentation on ash content is inconclusive: decrease from 4.8% to 2.3% (Hu et al. (2010); remain unchanged (Shrestha & Noomhorm, 2001); and increase from 5.0 to 5.6%–7.4% (Sarkar, Jones, Gore, Craven, & Somerset, 1994). Wei, Wolf‐Hall, and Chang (2001) reported that there is significant change in the level of mineral content. The initial ash content was 5.38% (DM), which significantly decreased with processing time and reached a minimum of 5.26% (DM) in the sample of soaked black soybeans. In contrast, a slight decrease in ash content was observed in black soybeans steamed for 35 min. With increase in fermentation time, the ash content of fermented beans inoculated with *B*. *natto* decreased gradually, and the ash content of fermented black soybeans was significantly lower than the samples of soaked and steamed black soybeans after 48‐hr fermentation.

Slight decrease in crude fiber content was noticed in all the varieties of *natto* in comparison with the respective raw materials (Table 6). According to Kedar (1994), crude fiber content decreased significantly during *kinema* fermentation by *Bacillus subtilis* and this decrease is governed by the enzymatic activity of the *natto*‐fermenting organism. Crude fiber level of soybeans also decreased during fermentation due to hydrolysis by carbohydrate‐splitting enzymes from *B. subtilis* (Shrestha & Noomhorm, 2001).

There was decrease in carbohydrate in all the varieties of *natto*. Although the sugar content of a soybean is only 4%–6%, it is important as a key constituent for umami. During soaking and steaming, the oligosaccharides of soybeans are partially lost in soaking water or cooking drain water and a part of them are decomposed. During fermentation, sucrose decreased to about one‐seventh, and both raffinose and stachyose decreased to about one‐third of the starting levels within 10 hr after starting fermentation. Taira, Tanaka, Saito, and Saito (1990) reported that soybeans with a high free‐sugar content, especially with high raffinose and stachyose content, are appropriate for *natto*. They deduced that sucrose is easily degraded by *natto* bacilli during fermentation, and there is a possibility that fermentation would stop too soon if the sucrose content is high, but raffinose and stachyose are difficult to degrade by the organisms and the fermentation would proceed adequately if they are present. According to Hayashi (1959), fermentable carbohydrate of soybean almost totally disappears in 24 hr of fermentation.

There is increase in pH after fermentation of soybean. The release of ammonia during fermentation increases the pH from almost neutral to 8 (Karki, 1986). This shows that there was equivalent amount of ammonia production during fermentation in all the varieties of *natto*. The pH of the steamed beans was slightly acidic (pH 6.2–6.8) and that *natto* products were slightly alkaline (pH 7.2–7.6) (Wei et al., 2001).

Calcium content and iron content of all the varieties of *natto* were higher than their respective raw materials (Table 6) of white, black, and brown soybean *natto*. Nikkuni et al. (1995) have reported the increment of calcium of raw soybean to *natto* formation (186–281) mg/100 g, respectively. The data represented in Table 9 showed that the microbiological analysis of *natto* contained lower TPC as compared to 6.4 × 10^2^ cfu/g. Yeast and mold count were found in the range of lower than 100.

## 4 CONCLUSIONS

*Natto* produced from pure culture as it gives less odor than kinema which has been highly objectionable for many consumers and can be preferred as it contains more protein. The technology has been so devised and developed so as to optimally suite the Nepalese status. Hence, the technology should be so evolved as to make it more appealing to the masses without much affecting its cost. An attractive packaging aided with strong promotional activities could surely help in establishing *natto* as a household name and projecting it as a cosmopolitan commodity. It develops the methods to make *natto* more acceptable among the larger part of the population because of its low content of flavor and odor. It is a cost‐effective, high‐quality ingredient that can replace dairy, egg, and meat proteins as consumers search for ever‐increasing variations to diet staples.

## CONFLICT OF INTEREST

The authors declare that they do not have any conflict of interest.

## ETHICAL STATEMENT

In this research work, I solely confirm that there has been no harm to human and animal. I testify solely that this article is my own work; there are no other co‐authors. The manuscript is not currently being considered for publication in another journal.
